# Book Reviews

**Published:** 1800-02

**Authors:** 


### MEDICINE AND SURGERY.

*Effays on the Venereal Difeafe and its concomitant Affections; Part the Second; containing additional Evidence, with Critical and Practical Remarks, on the new Saline Antifyphilitic Remedies, and an Anfwer to fome Objections made againft the former Part.* By WILLIAM BLAIR, A. M. F. M. S. Surgeon of the Lock Hofpital and Afylum, and of the Finfbury Difpenfary, &c. 8vo. pp. 352. Six fhillings in boards, 1799. London: SYMONDS, JOHNSON, &c.

The author appears to us, to have collected the facts with great diligence and perfeverance, while he has exhibited them with commendable impartiality. His general inference is, that the new remedies cannot be relied upon for the *radical* cure of the *fecondary* fymptoms of fyphilis, but that the nitrous or nitric acid, given "in conjunction or alternately with mercury," may be highly ufeful in promoting the cure. The cafes in which the acid proves moft beneficial, are pointed out, as well as the beft method of employing it.

[ To be continued in our next. ]

*An*

*An Essay on the Means of lessening the Effects of Fire on the Human Body. By* James Earle, Esq. Surgeon Extraordinary to the King, and to his Majesty's Household, and senior Surgeon to St. Bartholomew's Hospital, 8vo. pp. 44. with a plate, 1799. London. Johnson.

The means recommended by the author, consist in the speedy application of cold water, or water cooled by ice, which is to be renewed as often as it becomes warm. The cuticle of the burnt or scalded part is not to be removed; all stimulant and oily applications are to be avoided. By these means, the cure will be accomplished without leaving scars or lameness of the part.

"Since I discovered the advantage arising from ice," says Mr. Earle, "I have had many opportunities of using it on large and extensive burns, which have served to confirm me in my good opinion of its beneficial effects, whenever it has been timely and properly applied. In several cases it has happened that, either from motives of delicacy on the part of the patient, or from the attention of every one concerned being occupied with the most apparent injuries, parts which were burnt, have not been discovered in time to receive benefit from the cold application, in consequence of which the cuticle in those parts is separated, sloughs have formed and have been cast off, leaving sores difficult to be healed; while the parts in their neighbourhood, more severely burnt, but covered with ice, have escaped without a blemish.

"I could detail many instances of mischief prevented, and cures effected by these means; but as the progress of them was in general similar to the two cases already mentioned, it does not appear to be necessary to add to their testimony.

"I shall, however, take notice of one which occurred lately. A gentleman was much scalded by the overturning of a tea urn; I saw him soon after the accident, and sent to the nearest confectioner for ice, with which the burnt parts were bathed; the heat and pain were soon lessened, and afterwards he felt very little inconvenience.

"Whether modern philosophers will allow that fire applied to any part of the human body does remain united to it for a time, I am not certain; but such appears to me to be the fact; and this opinion has, I find, been maintained by several very respectable authors.

"It follows, then," according to the foregoing observations, "that the sooner the ice is applied after the accident has happened, the better, as the fire will have less time to do mischief. If the application be deferred till blisters are formed, and sloughs produced, a great degree of ease may be obtained; but the destruction of the parts which has already taken place, cannot entirely be prevented from going through its usual course of sloughs and suppuration.

---

*N. B. For want of room we have reluctantly been obliged to postpone our account of Dr.* Fothergill's "*Essay on the Preservation of Shipwrecked Mariners*; *and Dr.* Haygarth's *interesting Treatise on the Fictitious Tractors and Epidemical Convulsions*.

